# Supplementary material for: Charge state-dependent symmetry breaking of atomic defects in transition metal dichalcogenides
Source: Nat Commun. 2024 Mar 28;15:2738. doi: 10.1038/s41467-024-47039-4 (PMC11258346; doi:10.1038/s41467-024-47039-4)
Supplement: Supplementary file 1 — Supplementary Information [file 41467_2024_47039_MOESM1_ESM.pdf]

# Supplementary Information:
# Charge State-Dependent Symmetry Breaking of Atomic Defects in Transition Metal Dichalcogenides

Feifei Xiang,[1,*] Lysander Huberich,[1,*] Preston A. Vargas,[2,*] Riccardo Torsi,[3] Jonas Allerbeck,[1] Anne Marie Z. Tan,[2,4] Chengye Dong,[5] Pascal Ruffieux,[1] Roman Fasel,[1] Oliver Gröning,[1] Yu-Chuan Lin,[3,6] Richard G. Hennig,[2] Joshua A. Robinson,[3,5,7] and Bruno Schuler[1,†]

[1] *nanotech@surfaces Laboratory, Empa – Swiss Federal Laboratories for Materials Science and Technology, Dübendorf 8600, Switzerland*

[2] *Department of Materials Science and Engineering, University of Florida, Gainesville, FL, 32611, USA*

[3] *Department of Materials Science and Engineering, The Pennsylvania State University, University Park, PA 16082, USA*

[4] *Institute of High Performance Computing (IHPC), Agency for Science, Technology and Research (A*STAR), Singapore 138632, Republic of Singapore*

[5] *Two-Dimensional Crystal Consortium, The Pennsylvania State University, University Park, PA 16802, USA*

[6] *Department of Materials Science and Engineering, National Yang Ming Chiao Tung University, Hsinchu City 300, Taiwan*

[7] *Department of Chemistry and Department of Physics, The Pennsylvania State University, University Park, PA, 16802, USA*

(Dated: March 18, 2024)

## CONTENTS

Supplementary Figures 3

Supplementary Notes 19

Supplementary Experiments 19

Supplementary Calculations 20

Supplementary References 22

References 22

* These three authors contributed equally

† bruno.schuler@empa.ch

## SUPPLEMENTARY FIGURES

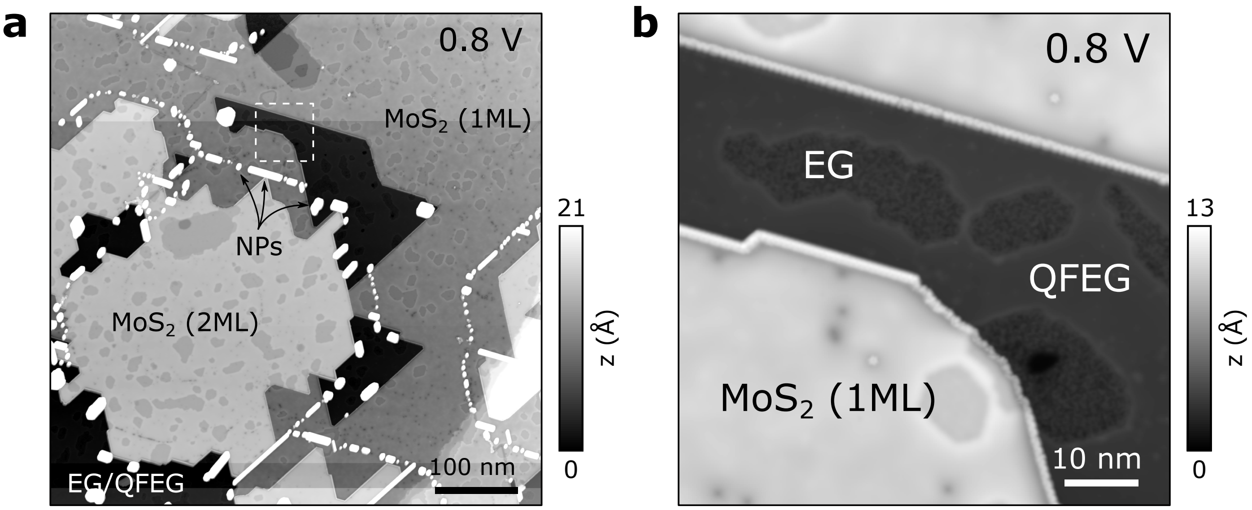


Figure 1. **Monolayer and bilayer $MoS_2$ on EG and QFEG. a**, Large-scale (800 nm × 800 nm) STM topography ($I = 50\,\mathrm{pA}$) of mono- and bilayer $MoS_2$ on mostly hydrogen-intercalated epitaxial graphene. **b**, Close-up of dashed rectangle in a, showing contrast difference between EG and QFEG areas, and how they appear if overgrown by $MoS_2$.

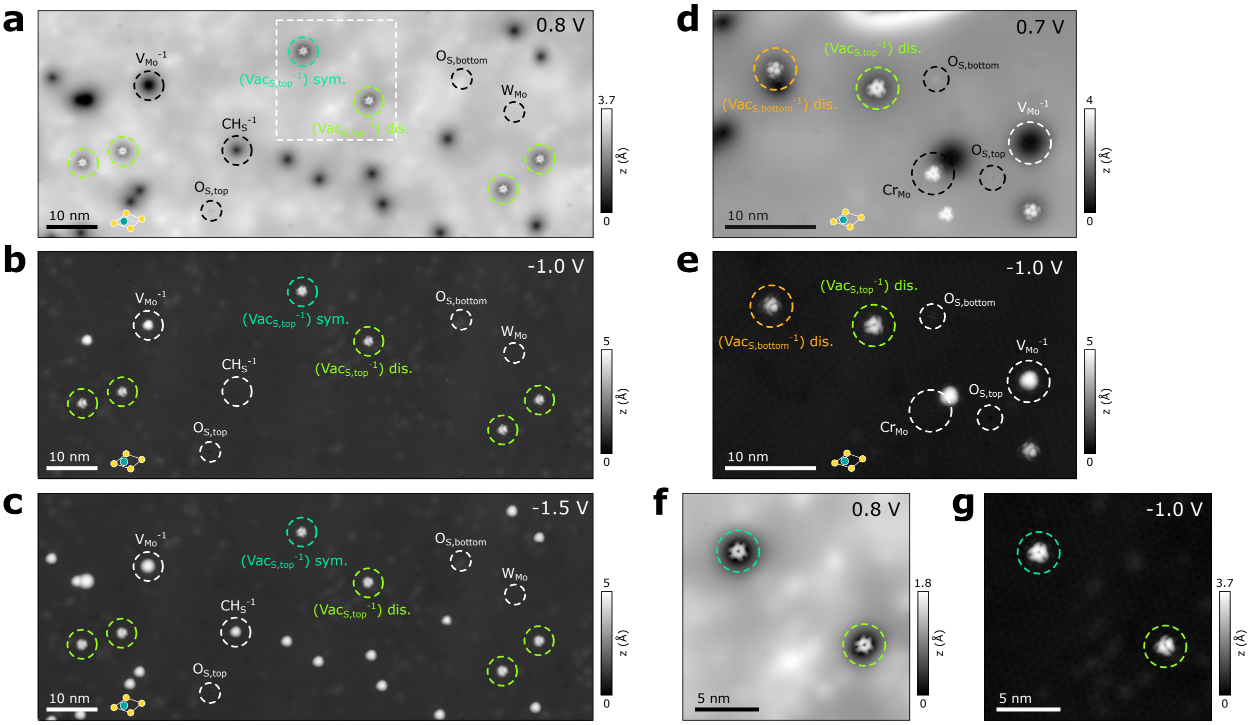


Figure 2. **Monolayer $MoS_2$/QFEG defects overview. a-g**, STM topographies ($I = 50\,$pA) of annealing-induced negatively charged sulfur vacancies ($Vac_S^{-1}$) (colored circles) in monolayer $MoS_2$/QFEG along with other as-grown defects such as $O_S$[1,2], $CH_S$[3], $V_{Mo}$[4], $Cr_{Mo}$[2], and $W_{Mo}$, labelled in black. The defect assignment is grounded in comprehensive characterization outlined in the referenced works. The STM contrast 100-200 mV above the TMD conduction band edge in conjunction with their unique STS fingerprint allow us to reliably assign defect types across various TMD semiconductors. Top and bottom $Vac_S^{-1}$ can be distinguished by their distinct STM contrast[5,6]. The symmetric and distorted $Vac_S^{-1}$ can be identified at negative bias voltage. A close-up of the dashed box in a is shown in f,g.

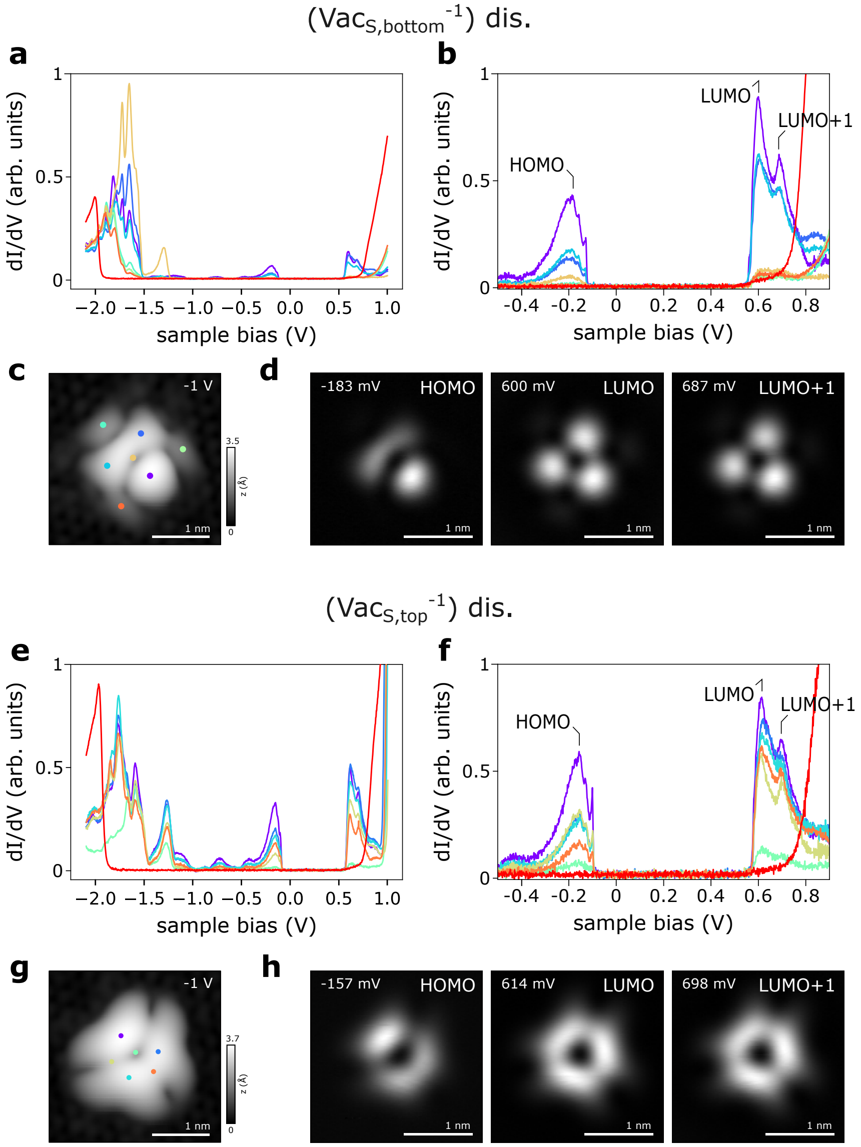


Figure 3. **STS of distorted $\mathbf{Vac_{S,top}^{-1}}$ and $\mathbf{Vac_{S,bottom}^{-1}}$.** **a,b**, $\mathrm{d}I/\mathrm{d}V$ spectra of a distorted $\mathrm{Vac_{S,bottom}^{-1}}$ at the locations indicated in c. The red spectrum is taken on the pristine $MoS_2$ surface. **c**, STM image of $\mathrm{Vac_{S,bottom}^{-1}}$. **d**, $\mathrm{d}I/\mathrm{d}V$ maps of the frontier orbitals labeled in b. **e,f**, $\mathrm{d}I/\mathrm{d}V$ spectra of a distorted $\mathrm{Vac_{S,top}^{-1}}$ at the locations indicated in g. The red spectrum is taken on the pristine $MoS_2$ surface. **g**, STM image of $\mathrm{Vac_{S,top}^{-1}}$. **h**, $\mathrm{d}I/\mathrm{d}V$ maps of the frontier orbitals labeled in f. The HOMO orbitals for both the bottom and top S vacancy resemble the same asymmetry as observed in the corresponding STM topography at negative bias. Note that the asymmetry of the $\mathrm{Vac_{S,top}^{-1}}$ HOMO shown in h is different from the one shown in Fig. 1e. Both orbital shapes are regularly observed and may indicate a different local strain profile as discussed in the main text. $V_{\mathrm{mod}}$ = 10 mV (a,e), 2 mV (b,f), and 20 mV (d,h).

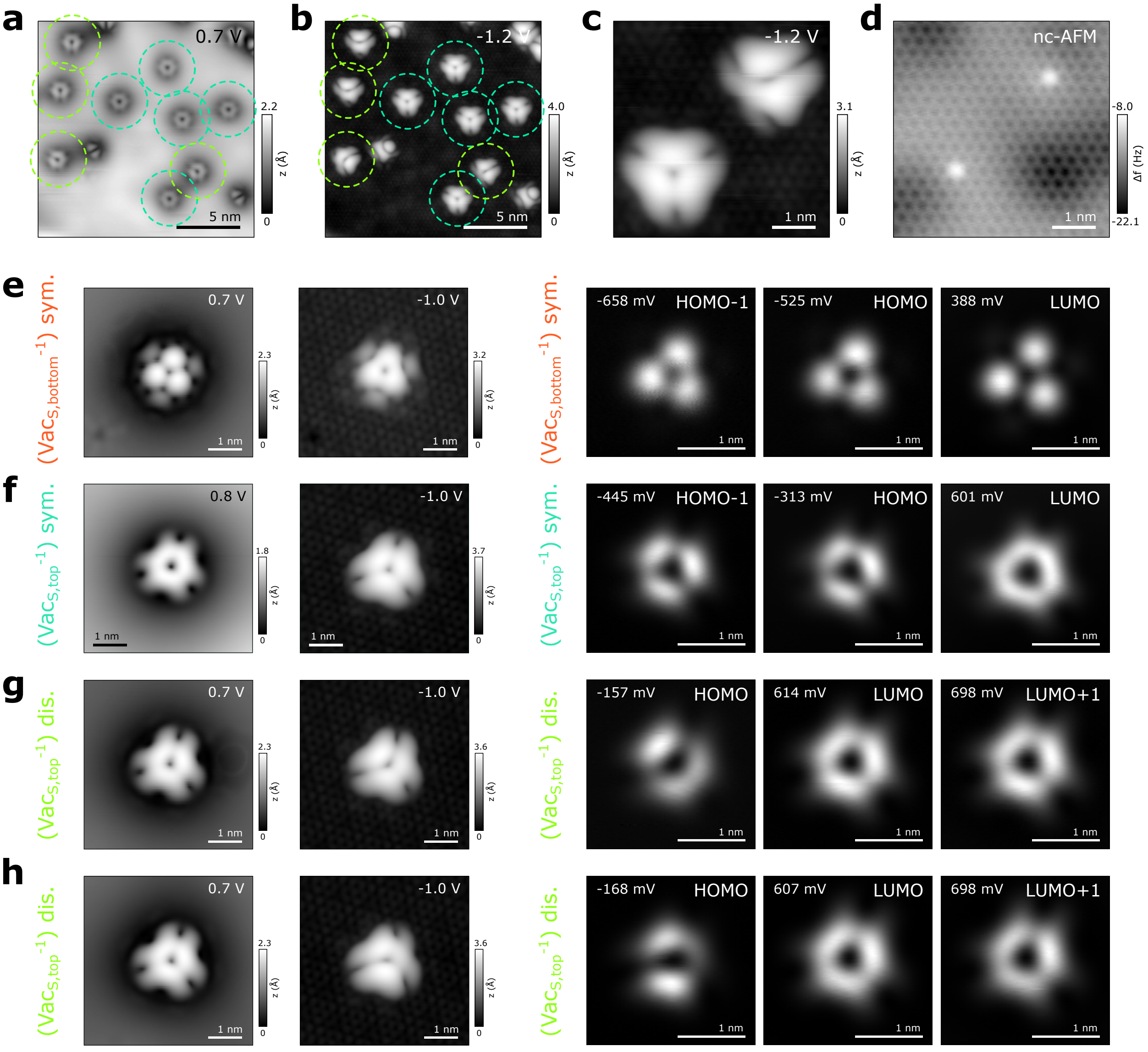


Figure 4. **Symmetric and distorted $\mathrm{Vac}_\mathrm{S}^{-1}$ comparison.** **a,b**, STM topography ($I = 50\,\mathrm{pA}$) of negatively charged sulfur top vacancies in $MoS_2$/QFEG at positive (a) and negative (b) sample bias. **c,d**, STM topography ($I = 50\,\mathrm{pA}$) and nc-AFM image (metal tip) of a symmetric and distorted $\mathrm{Vac}_{\mathrm{S,\,top}}^{-1}$. **e-h**, STM topographies ($I = 50\,\mathrm{pA}$) (left) and $\mathrm{d}I/\mathrm{d}V$ maps (right) of the symmetric $\mathrm{Vac}_{\mathrm{S,\,bottom}}^{-1}$ (e), symmetric $\mathrm{Vac}_{\mathrm{S,\,top}}^{-1}$ (f), and two versions of a distorted $\mathrm{Vac}_{\mathrm{S,\,top}}^{-1}$ (g,h) for comparison. Panels b-d, and h are the same as in Fig. 1a-c, and Fig. 1e, but are reprinted for comparison.

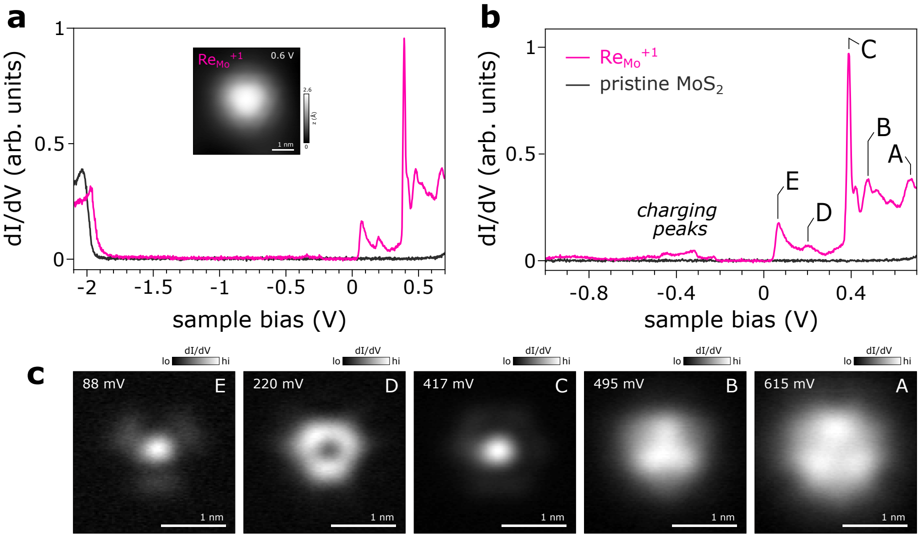


Figure 5. **dI/dV spectra and orbital imaging of $\mathbf{Re_{Mo}^{+1}}$ on monolayer $\mathbf{MoS_2}$/QFEG. a**,**b** dI/dV spectra of $Re_{Mo}^{+1}$ (pink) with the main defect resonances indicated, and reference spectrum on pristine $MoS_2$ in gray. In panel **b**, data is acquired at a reduced tip-sample distance, emphasizing frontier defect states within a smaller bias window. **c**, Constant height dI/dV maps of the defect orbitals labelled in a and b. Lock-in modulation: $V_{mod}$ = 20 mV (a and c) and $V_{mod}$ = 10 mV (b).

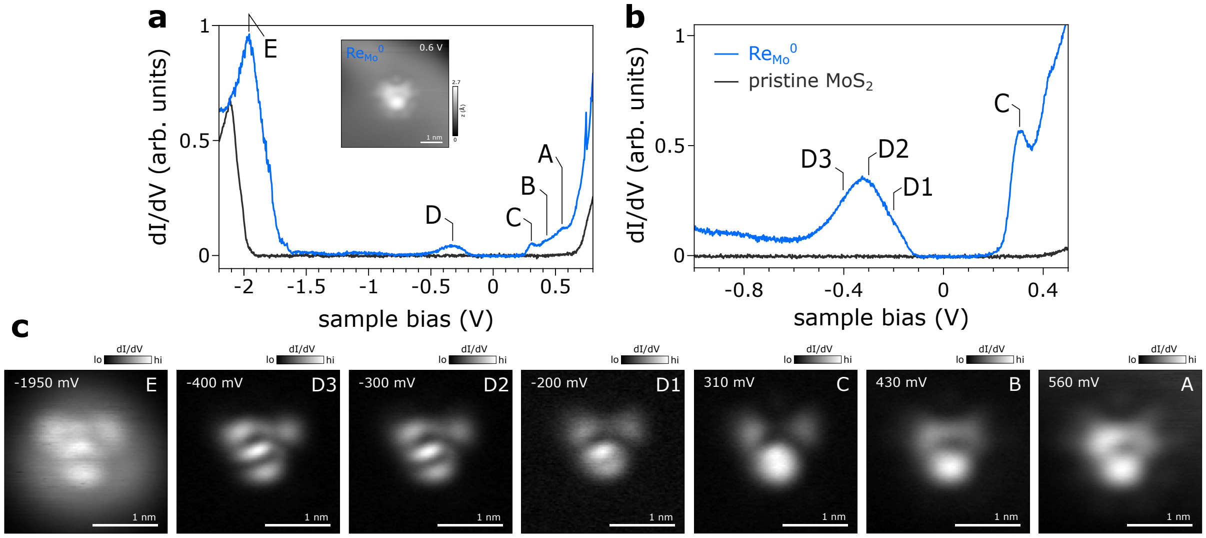


Figure 6. **dI/dV spectra and orbital imaging of $Re_{Mo}^0$ on monolayer $MoS_2$/QFEG. a**,**b** dI/dV spectra of $Re_{Mo}^0$ (blue) with the main defect resonances indicated, and reference spectrum on pristine $MoS_2$ in gray. In panel **b**, data is acquired at a reduced tip–sample distance, emphasizing frontier defect states within a smaller bias window. **c**, Constant height dI/dV maps of the defect orbitals labelled in a and b. Note that the defect state evolution of the resonance D proceeds along a different crystallographic direction as compared to Fig. 5 in the main text. Lock-in modulation: $V_{mod}$ = 20 mV (a and c) and $V_{mod}$ = 10 mV (b)

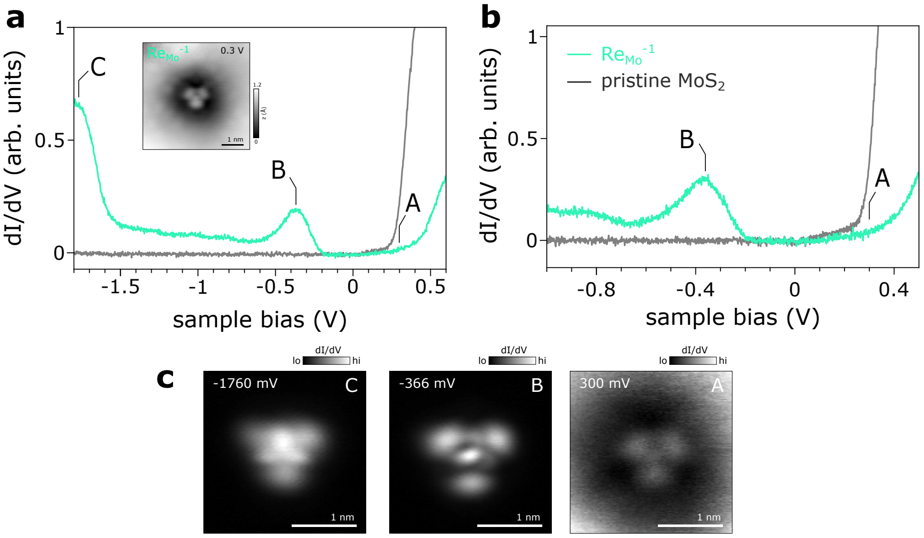


Figure 7. **dI/dV spectra and orbital imaging of $Re_{Mo}^{-1}$ on monolayer $MoS_2$/EG. a**,**b** dI/dV spectra of $Re_{Mo}^{-1}$ (green) with the main defect resonances indicated, and reference spectrum on pristine $MoS_2$ in gray. In panel **b**, data is acquired at a reduced tip–sample distance, emphasizing frontier defect states within a smaller bias window. **c**, Constant height dI/dV maps of the defect orbitals labelled in a and b. Note that the defect state evolution as discussed in the main text is not shown here. Lock-in modulation: $V_{mod}$ = 20 mV (a and c) and $V_{mod}$ = 10 mV (b)

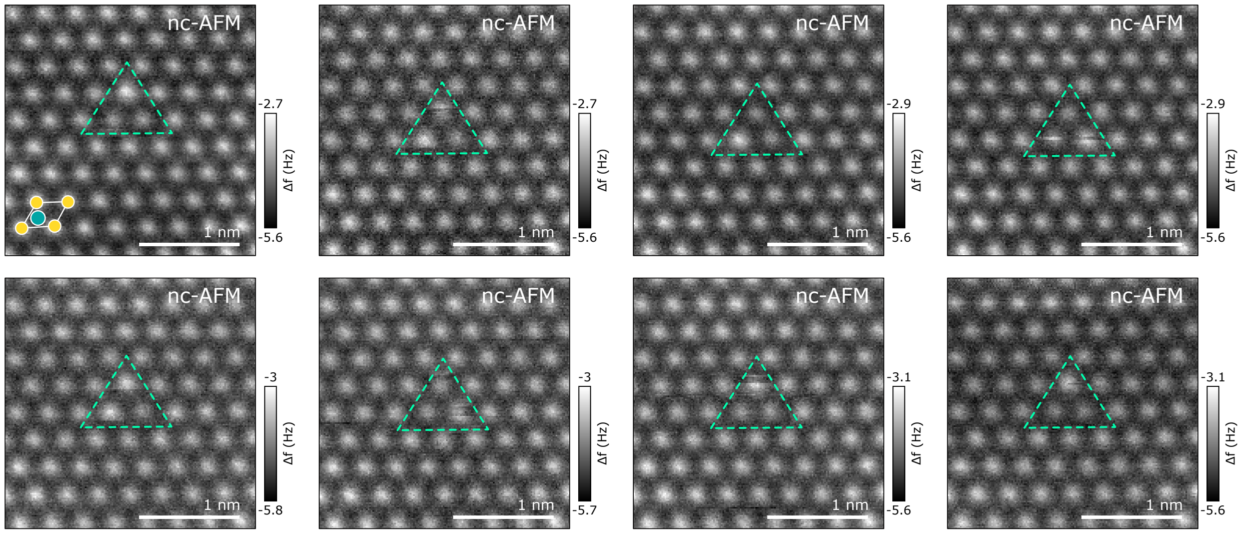


Figure 8. **Conformational switching of $\mathbf{Re_{Mo}^{-1}}$.** Consecutive CO-tip nc-AFM images of the same $Re_{Mo}^{-1}$ impurity. Top sulfur atoms are repulsive (bright) and molybdenum atoms attractive (dark). The $MoS_2$ unit cell is indicated at the bottom left of the first image and the position of the Re impurity is highlighted by the green triangle. One of the three neighboring top S atoms next to each Re impurity is protruding out of plane (brightest atom). Within or in-between consecutive images the protruding S atom is switched between one of the three equivalent lattice sites.

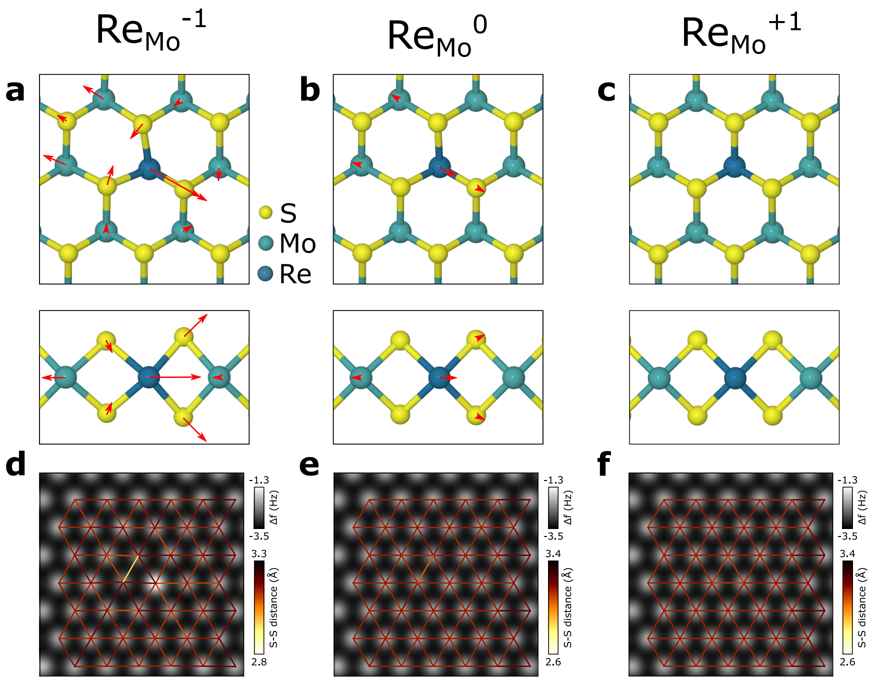


Figure 9. **Calculated geometry of $\mathbf{Re_{Mo}}$ in different charge states. a-c**, Ground state geometry of $Re_{Mo}$ in the -1 $e$ (a), 0 $e$ (b), and +1 $e$ (c) charge state, respectively. The red arrows depict the atom relaxation as compared to the pristine $MoS_2$ lattice. The arrows are ten times longer than the actual relaxation. Both $Re_{Mo}^{-1}$ and $Re_{Mo}^{0}$ exhibit considerable Jahn-Teller distortions, while $Re_{Mo}^{+1}$ preserves a symmetric lattice geometry. **d-f**, Simulated CO-tip nc-AFM images of the defect geometries in a-c. The $\Delta f$ contrast with one protruding S atom, adjacent to the Re impurity for $Re_{Mo}^{-1}$ and to a lesser degree for $Re_{Mo}^{0}$ is in excellent agreement with experiment (see Fig. 3). The apparent sulfur-sulfur distances in the top S layer is indicated by the red-to-yellow lines. Significant lateral compressive strain introduced by the $Re_{Mo}^{-1}$ impurity is seen at nearest-neighbor S atoms opposite to the protruding S atom.

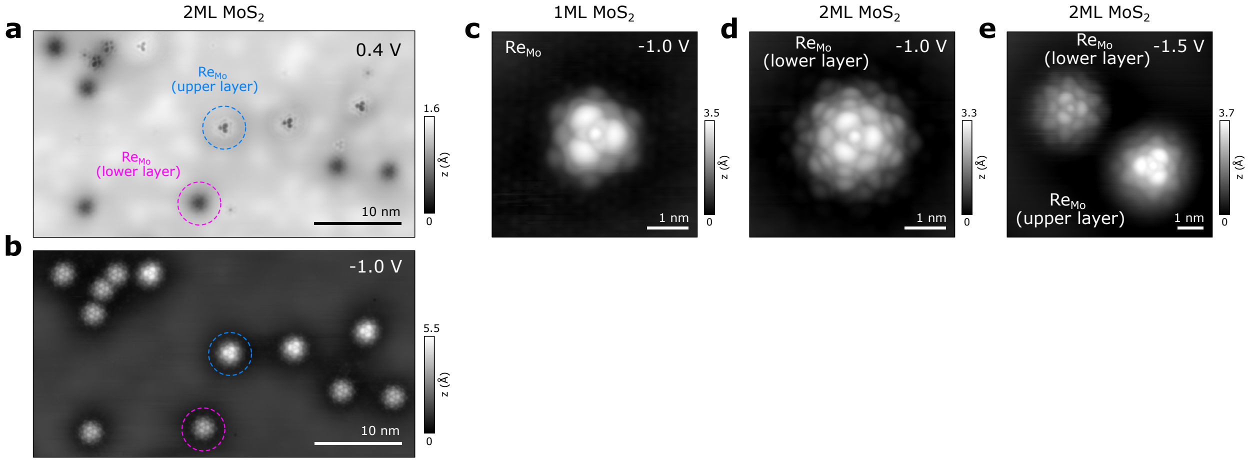


Figure 10. **STM contrast of $Re_{Mo}$ on bilayer $MoS_2$. a, b**, STM topography ($I = 50$ pA) of Re impurities in bilayer $MoS_2$ on EG scanned at 0.4 V and -1 V, respectively. **c, d**, STM orbital image of $Re_{Mo}$ in monolayer and bilayer $MoS_2$. The $Re_{Mo}$ in d is located in the lower $MoS_2$ layer. **e** STM orbital image of two $Re_{Mo}$ in bilayer $MoS_2$. The upper left impurity is located in the lower $MoS_2$ layer and the lower right impurity in the upper $MoS_2$ layer, with a brighter and mirrored appearance, as expected for 2H stacking.

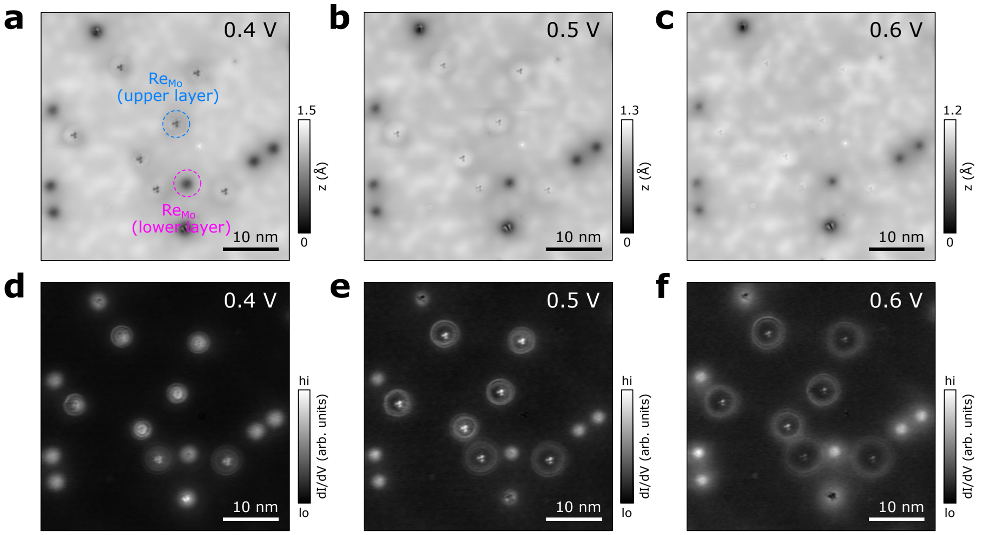


Figure 11. **Tip-induced charging rings at Re impurities in bilayer $MoS_2$. a-c**, STM topographies ($I = 50$ pA) of Re impurities in the upper and lower $MoS_2$ layer grown on EG imaged at 0.4 V (a), 0.5 V (b), and 0.6 V (c), respectively. **d-f**, $dI/dV$ channel recorded simultaneously with the STM topographies a-c ($V_{mod} = 20$ mV).

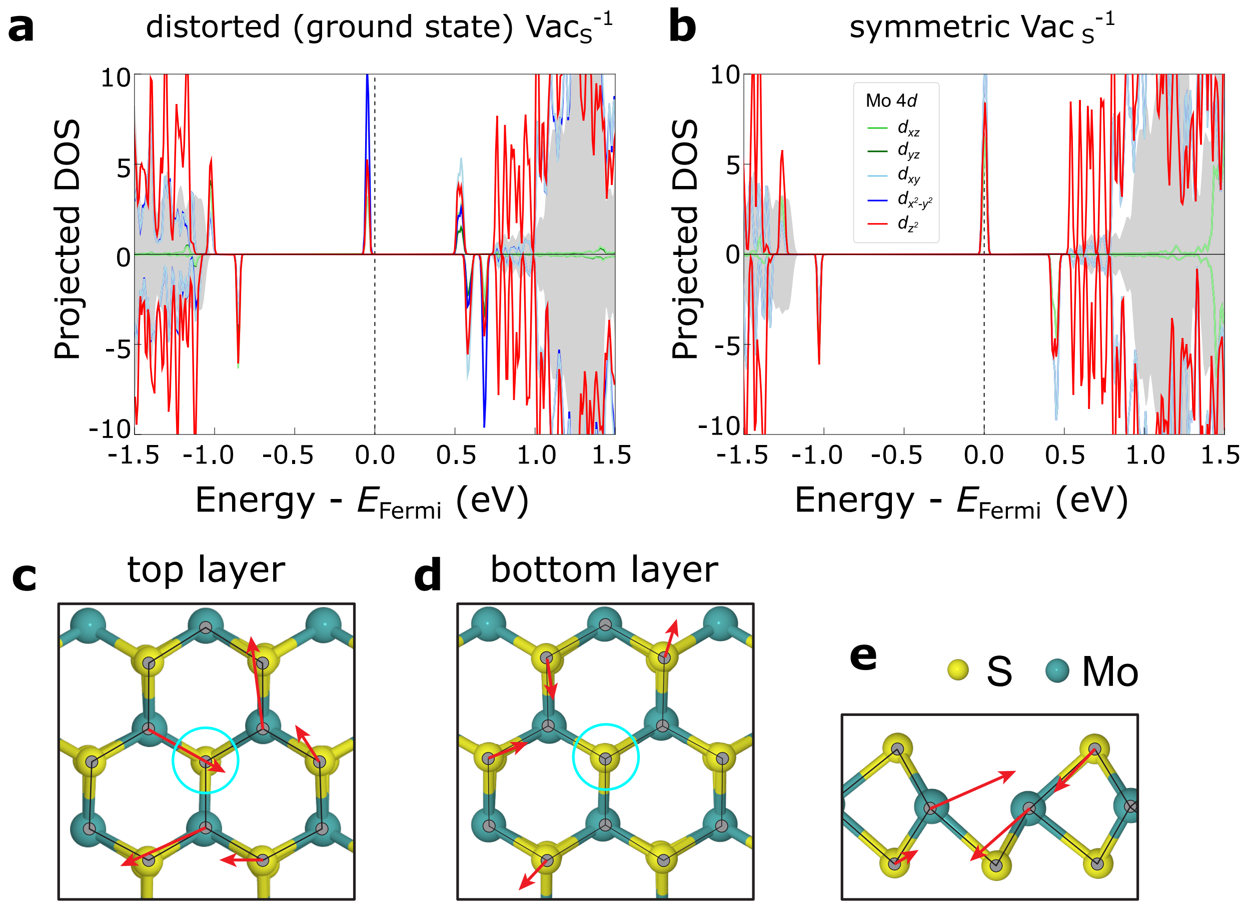


Figure 12. **Projected density of states of the distorted and symmetric $Vac_S^{-1}$ in $MoS_2$.** **a**, $Vac_S^{-1}$ in the distorted ground state geometry calculated in a 5×5 supercell. The density of states is projected onto the Mo $d$ orbitals. **b**, $Vac_S^{-1}$ in the constrained symmetric geometry (+142 meV ) **c**-**e**, calculated geometry of $MoS_2$ with a distorted $Vac_S^{-1}$, looking from the top layer (c), bottom layer (d) and side (e). The red arrows show the atom relaxations compared to the $MoS_2$ lattice with a symmetric $Vac_S^{-1}$ (atom positions are shown as gray ball and stick model). The position of $Vac_S^{-1}$ is located on the top layer of $MoS_2$ and is marked by a cyan circle. The arrow length is 50 times longer than the actual relaxation distance.

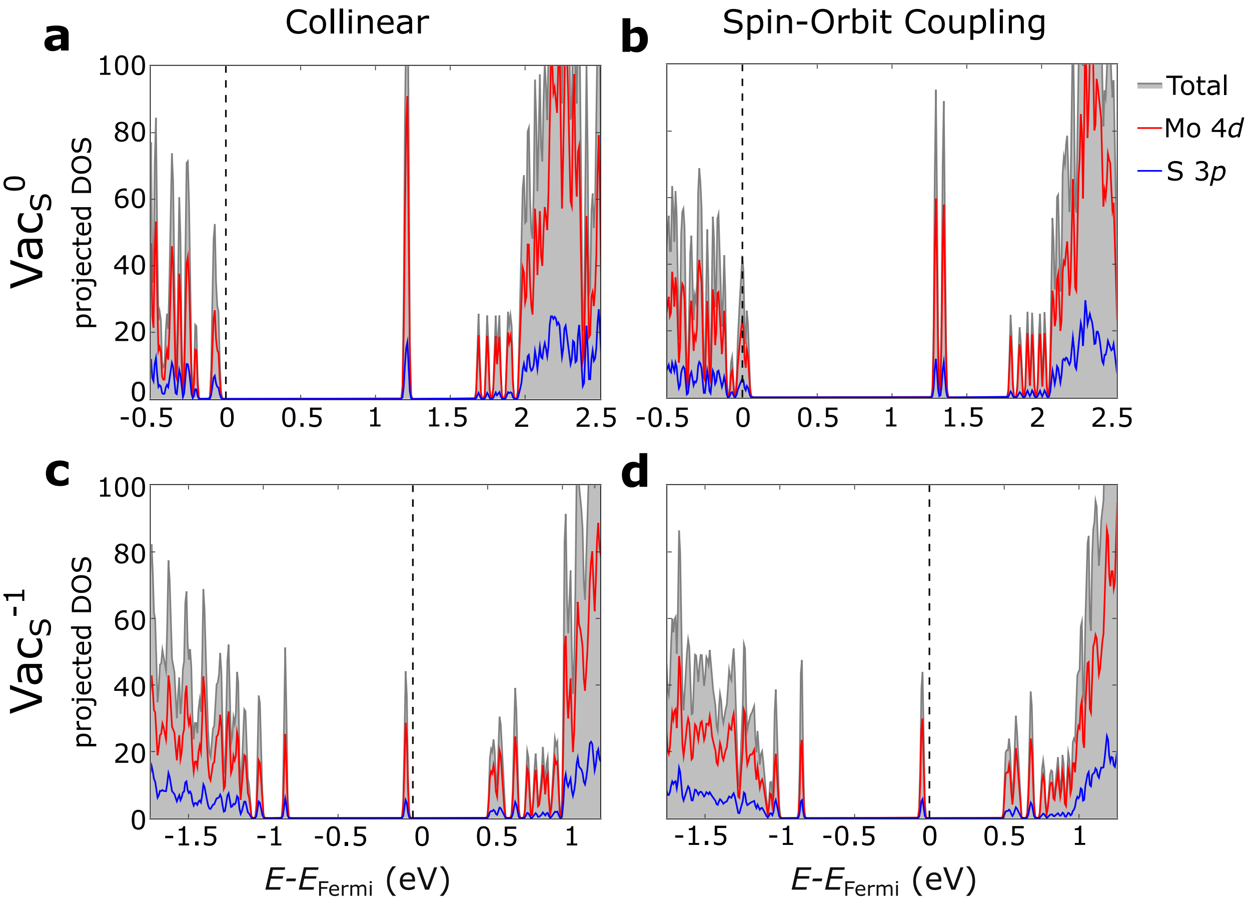


Figure 13. **Effect of spin-orbit coupling on $Vac_S$ defect states and symmetry breaking. a,b**, Projected density of states of $Vac_S^0$ in $MoS_2$ with and without considering SOC. **c,d**, Projected density of states of $Vac_S^{-1}$ in $MoS_2$ with and without considering SOC.

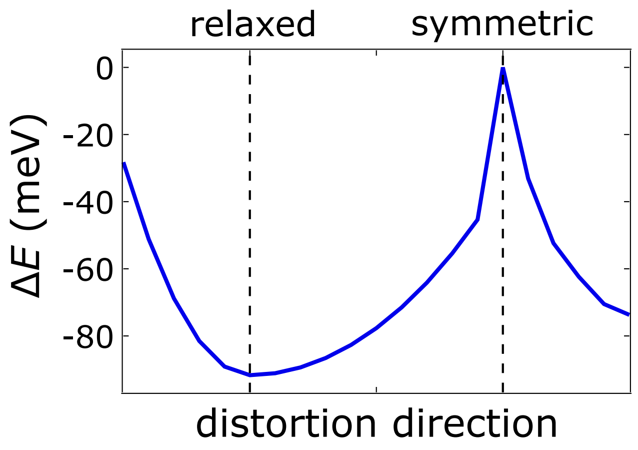


Figure 14. **Energy differences on a linearly interpolated path along the distortion coordinates for $Vac_S{}^{-1}$.**

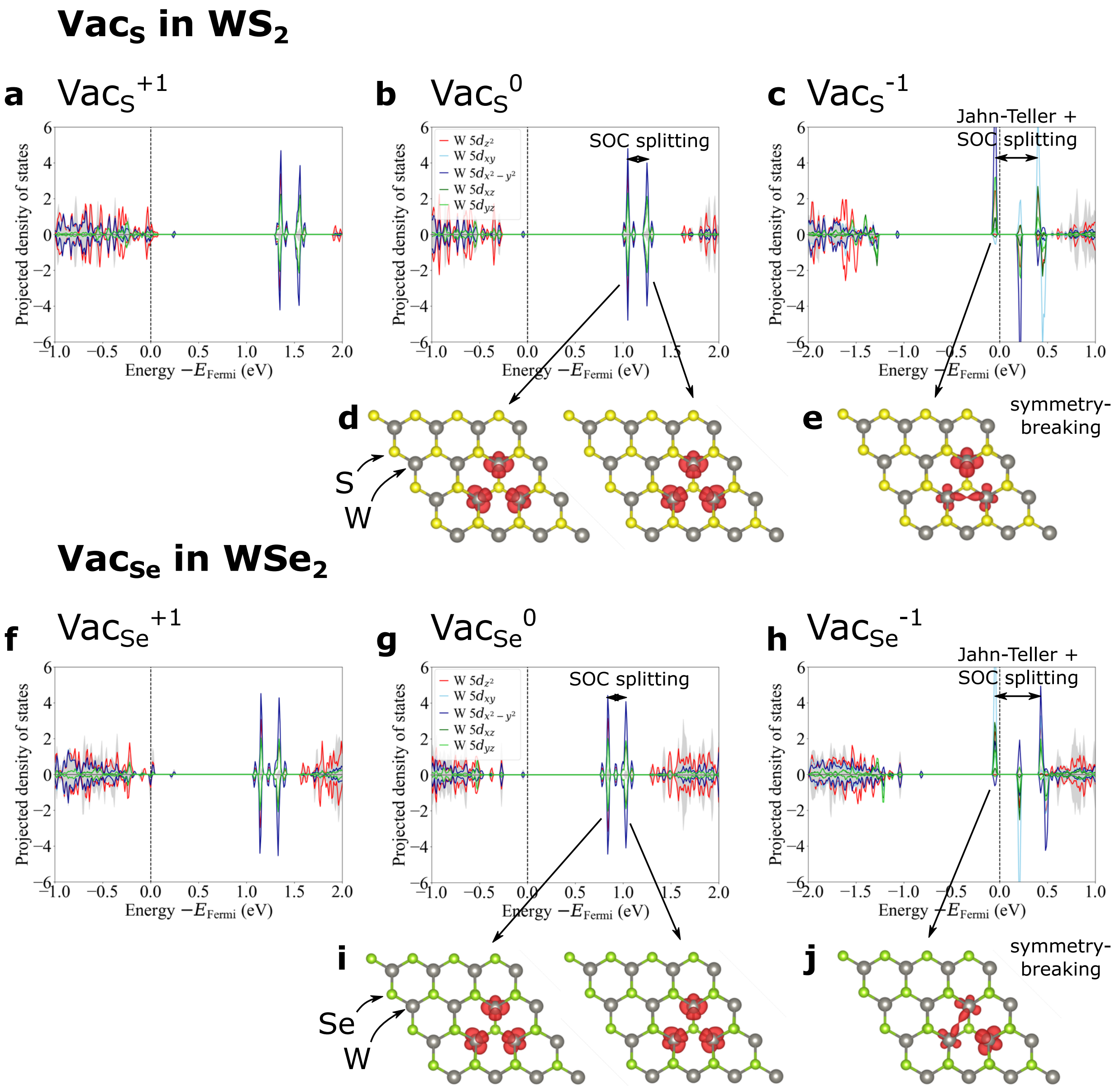


Figure 15. **Jahn-Teller effect for the negatively charged S vacancy in $WS_2$ and Se vacancy in $WSe_2$.** **a-c**, Projected density of states of $Vac_S$ in $WS_2$ in the +1, 0, and -1 charge state. $Vac_S^{-1}$ exhibits a symmetry-broken HOMO, shown in **e**, whereas the $Vac_S^0$ frontier orbitals, shown in **d**, are symmetric. **f-h**, Projected density of states of $Vac_{Se}$ in $WSe_2$ in the +1, 0, and -1 charge state. $Vac_{Se}^{-1}$ exhibits a symmetry-broken HOMO, shown in **j**, whereas the $Vac_{Se}^0$ frontier orbitals, shown in **i**, are symmetric.

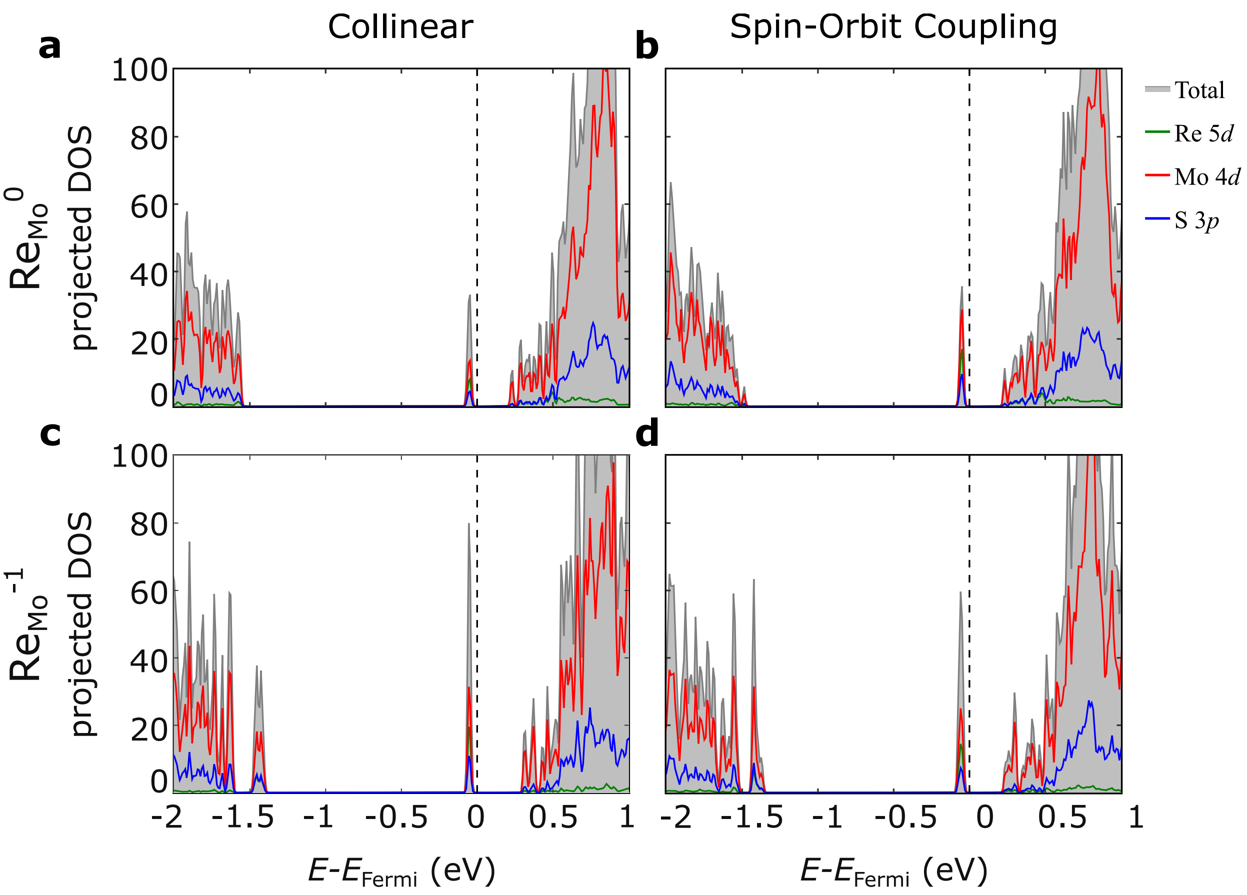


Figure 16. **Effect of spin-orbit coupling on $Re_{Mo}$ defect states and symmetry breaking. a,b**, Projected density of states of $Re^0_{Mo}$ in $MoS_2$ with and without considering SOC. **c,d**, Projected density of states of $Re^{-1}_{Mo}$ in $MoS_2$ with and without considering SOC.

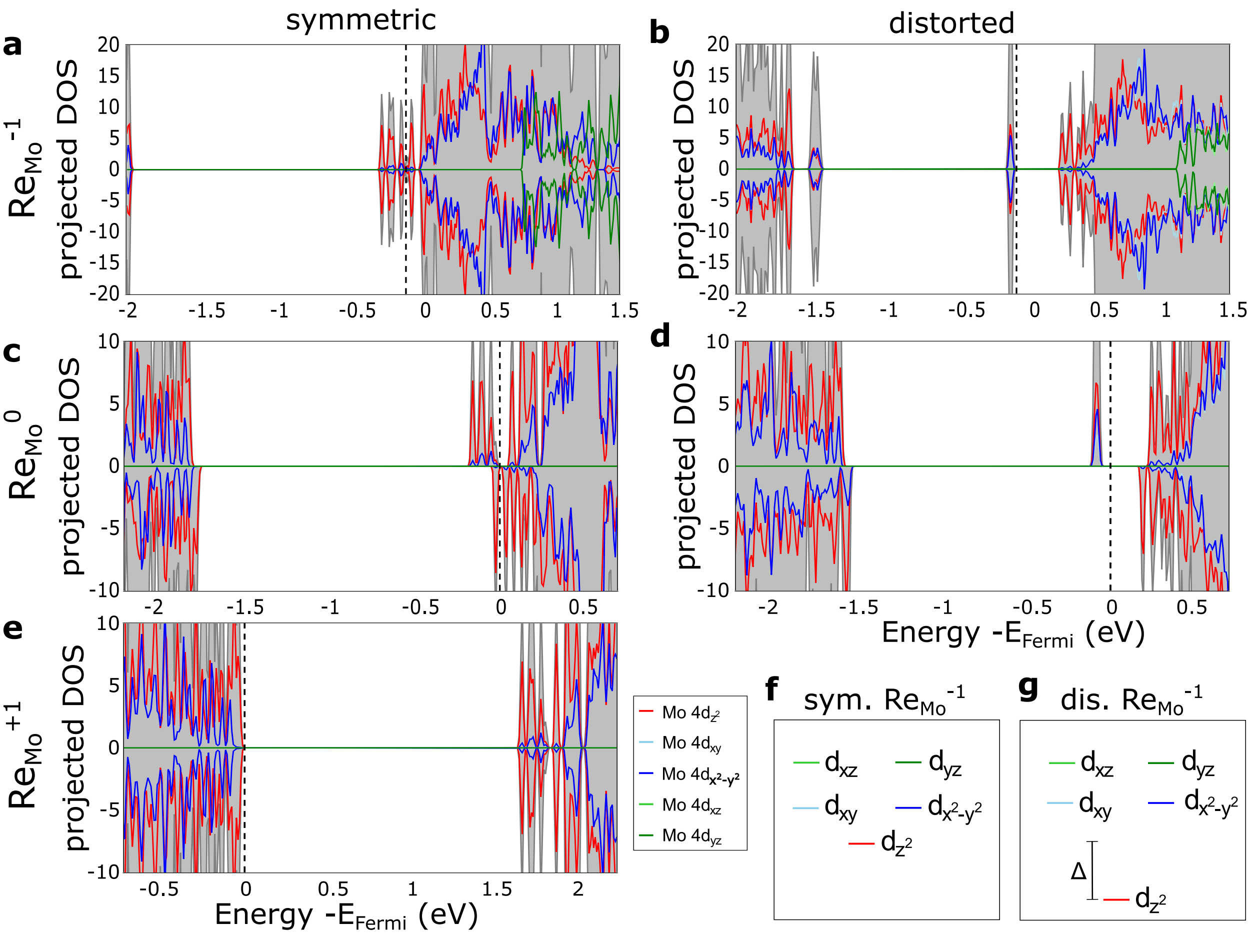


Figure 17. **Projected density of states of $Re_{Mo}$ in different charge states and geometries. a,b**, calculated pDOS of $Re_{Mo}^{-1}$ in symmetric (a) and distorted (b) configuration. **c,d**, calculated pDOS of $Re_{Mo}^{0}$ in symmetric (c) and distorted (d) configuration. For $Re_{Mo}^{-1}$ and $Re_{Mo}^{0}$ the distorted configuration is the ground state. **e**, calculated pDOS of $Re_{Mo}^{+1}$ in symmetric configuration. **f,g**, energy level diagrams of $Re_{Mo}^{-1}$ *d* orbitals in symmetric (f) and distorted (g) configuration.

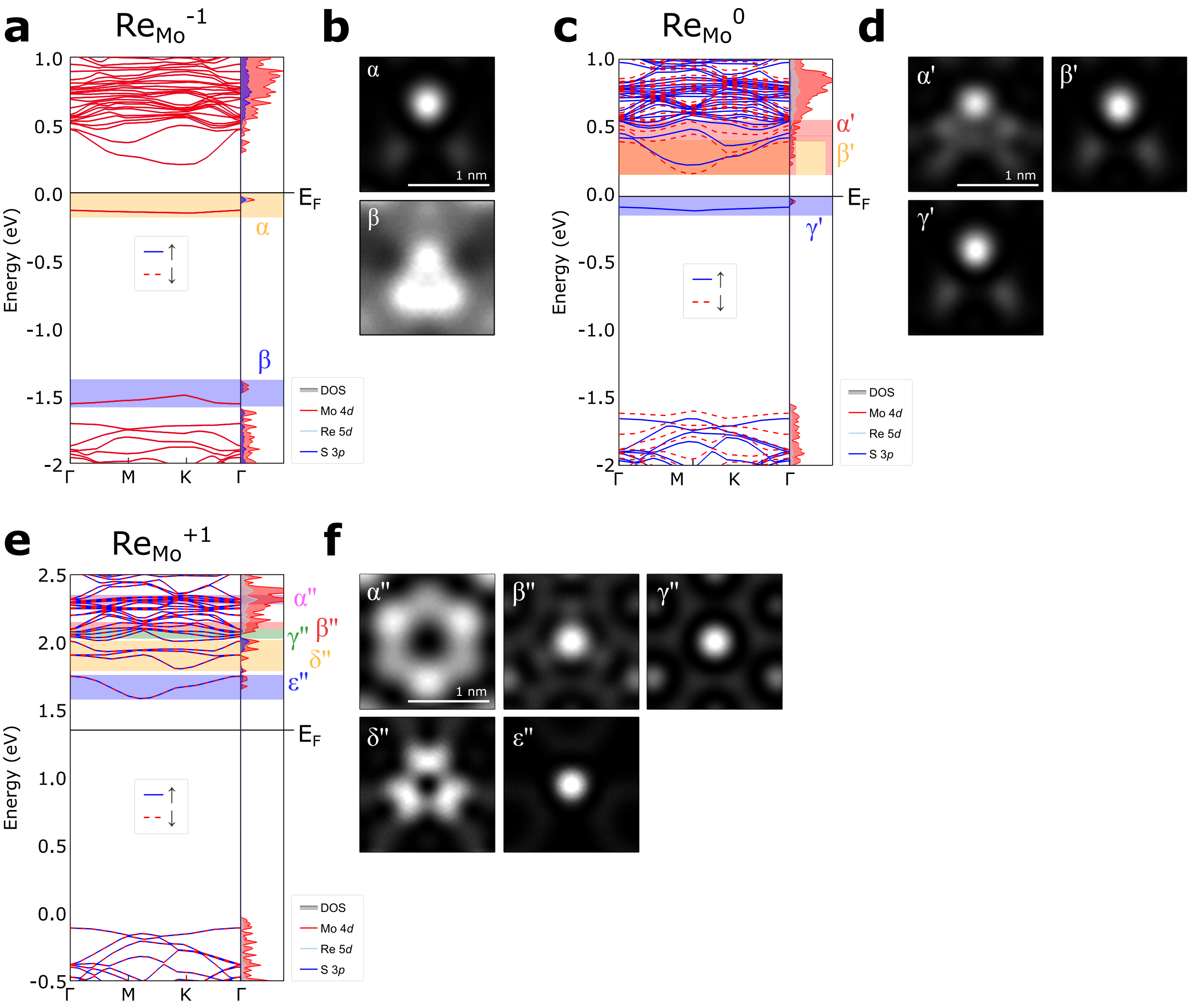


Figure 18. **DFT calculated band structures, projected density of states (pDOS) and simulated d$I$/d$V$ maps of $Re_{Mo}$ in different charge states. a,c,e**, band structure (left) and pDOS (right) of $Re_{Mo}^{-1}$ (a), $Re_{Mo}^{0}$ (c), and $Re_{Mo}^{+1}$ (e). **b** simulated dI/dV maps of $Re_{Mo}^{-1}$ at the corresponding energy marked in (a). **d** simulated dI/dV maps of $Re_{Mo}^{0}$ at the corresponding energy marked in (c). **f** simulated dI/dV maps of $Re_{Mo}^{+1}$ at the corresponding energy marked in (e).

## SUPPLEMENTARY NOTES

### Supplementary Experiments

More detailed conductance measurements of $\mathrm{Vac}_\mathrm{S}^{-1}$ are shown in Supplementary Figure 3. We measured the conductance ($\mathrm{d}I/\mathrm{d}V$) at several positions on each defect, the corresponding dI/dV spectra are shown in Supplementary Figure 3a, b, e, f, respectively. Apart from the frontier orbitals around zero bias, several hydrogenic bound states due to the negative charge can be identified above the valence band edge[7] (Supplementary Figure 3a and e). If we just focus on the frontier orbitals of both the distorted $\mathrm{Vac}_\mathrm{S,bottom}^{-1}$ and $\mathrm{Vac}_\mathrm{S,top}^{-1}$ (Supplementary Figure 3b and f), three resonances are revealed with with several vibronic excitations visible. As seen in the constant height dI/dV maps in Supplementary Figure 3d and h, the highest occupied defect resonance (HOMO) exhibits a clear two-fold symmetric shape, whereas the two unoccupied orbitals appear basically three-fold symmetric.

At positive bias, all $\mathrm{Vac}_\mathrm{S,\,top}^{-1}$ appear threefold symmetric, while at negative bias some appear threefold symmetric (turquoise circles in Supplementary Figure 2 and Supplementary Figure 4b), while other appear at reduced twofold symmetry (light green circle) at about an equal share. The high resolution STM images of symmetric and distorted $\mathrm{Vac}_\mathrm{S}^{-1}$ and the corresponding $\mathrm{d}I/\mathrm{d}V$ maps taken in constant height mode are displayed in Supplementary Figure 4e-h. The orbital symmetry breaking is most clearly revealed in the defect's HOMO orbital (cf. Supplementary Figure 4g,h).

$\mathrm{Re_{Mo}}$ on bilayer $\mathrm{MoS_2}$ on EG/SiC is shown in Supplementary Figure 10. Two types of $\mathrm{Re_{Mo}}$ defects can be found on the surface: $\mathrm{Re_{Mo}}$ in the lower layer $\mathrm{MoS_2}$ (marked by pink circle in Supplementary Figure 10a and b) and $\mathrm{Re_{Mo}}$ in the upper layer (marked by blue circle in Supplementary Figure 10a and b). Strikingly, the the upper and lower layer $\mathrm{Re_{Mo}}$ in 2ML $\mathrm{MoS_2}$ exhibits an interference pattern around the defect, which is not seen for monolayer $\mathrm{MoS_2}$ (cf. Supplementary Figure 10c and d). The interference pattern may originate from quasi-particle scattering of dispersive $\mathrm{MoS_2}$ bilayer states. In bilayer $\mathrm{MoS_2}$, the $\mathrm{Re_{Mo}}$ of the lower and upper layer are mirrored as expected for the 2H stacking (see Supplementary Figure 10e).

At positive sample bias, charging rings around the Re impurities that are induced by the tip electric field could be observed (Supplementary Figure 11). Such rings only appear for $\mathrm{Re_{Mo}}$ in the upper $\mathrm{MoS_2}$ layer. Re impurities in the lower layer appear as dark protrusions similar to on 1ML

$MoS_2$/EG. Re impurities in the upper layer, however, have a dark tri-lobal contrast surrounded by two concentric bright ring (Supplementary Figure 11a-c). These concentric rings can be best seen in d$I$/d$V$ maps recorded in constant current mode, shown in Supplementary Figure 11d-f. As expected the ring size increases with increasing bias. Presumably, the $Re_{Mo}$ in the upper layer are initially charge neutral but a small tip-electric field can already decharge them. Inside the charging rings, the defect is therefore negatively charged and the dark contrast corresponds to the depopulated defect orbital.

**Supplementary Calculations**

*Sulfur vacancy in $MoS_2$*

The DFT calculated projected density of states (pDOS) of symmetric and distorted $Vac_S^{-1}$ are shown in Supplementary Figure 12a and b. In the symmetric $Vac_S^{-1}$, a single electron occupies the two degenerate orbitals at the Fermi level, generating a Jahn-Teller instability. The degenerate orbitals have a comparable contribution from the $d_{x^2-y^2}$ (dark blue) and $d_{xy}$ (light blue) Mo orbital. In the distorted configuration, this degeneracy is lifted such that only a non-degenerate, fully occupied state exists with a predominant $d_{x^2-y^2}$ character (dark blue). The local JT displacements in the distorted geometry are shown in Supplementary Figure 12c-e. Mo atoms adjacent to the $Vac_S$ exhibit the largest displacemebts on the order of 5 pm compared to the symmetric case.

In Supplementary Figure 13 the projected density of states of $Vac_S$ in $MoS_2$ in the neutral and negative charge state with and without considering SOC is shown. As can be seen in Supplementary Figure 13b, SOC slightly lifts the degeneracy of the $Vac_S^0$ defect state. However, the splitting is rather small, as commonly observed for Mo $4d$ states. In the negative charge state, we observe the JT distortion with a very similar density of states with and without SOC, as seen in Supplementary Figure 13c and Supplementary Figure 13d, respectively. Therefore we can conclude that while effects of SOC are not quantitatively negligible, the system exhibits the same qualitative behavior with and without SOC.

In Supplementary Figure 14 the energy differences between the symmetric and distorted $Vac_S^{-1}$ structures are shown. The energy differences are referenced to the relaxed energy of the

symmetry-constrained structure. The fully relaxed structure has a considerably lower energy than the symmetric structure. The lack of a local minimum at the symmetric configuration is both notable and expected. The electronic structure in Supplementary Figure 12 clearly indicates a Jahn-Teller effect, which does not exhibit a local minimum in the potential energy surface at the symmetric state. Therefore, the symmetric configuration of $\mathrm{Vac}_\mathrm{S}^{-q}$ must be due to a different charge or spin state.

In Supplementary Figure 15 the projected DOS of a sulfur vacancy in $WS_2$ and selenium vacancy in $WSe_2$ is shown for different charge states. Despite the notably stronger SOC, a Jahn-Teller distortions is observed for the negative charge state, similar to $\mathrm{Vac}_\mathrm{S}^{-1}$ in $MoS_2$.

*Rhenium substitution in $MoS_2$*

In Supplementary Figure 16 the projected density of states of $\mathrm{Re}_\mathrm{Mo}$ in $MoS_2$ in the neutral and negative charge state with and without considering SOC is shown. In both cases, the effect of SOC is negligible. In particular, the pseudo-JT distortion is reproduced.

The DFT calculated pDOS of $\mathrm{Re}_\mathrm{Mo}$ in different charge states and geometries are shown in Supplementary Figure 17. For both the neutral and negative charge state a strong reconfiguration of the electronic structure of the frontier defect orbitals are observed upon distortion. The distortion leads to a net energy gain of the total energy of the system. The initial symmetric geometry shows an orbital degeneracy between $d_{xy}$, $d_{x^2-y^2}$ and $d_{xz}$, $d_{yz}$ in the unoccupied spectrum. The occupied defect orbital in the $\mathrm{Re}_\mathrm{Mo}^{-1}$ and $\mathrm{Re}_\mathrm{Mo}^{0}$ states is well projected onto the Re $5d_{z^2}$ orbital, which is not degenerate with any other states. Therefore, a regular Jahn-Teller effect cannot appear. However, we do see the $d_{z^2}$ lower in energy upon occupation. Therefore we propose a pseudo Jahn-Teller (pseudo-JTE) effect to be primarily responsible for the distortion of the defect.

In the case of the positively charged defect, there is no distorted structure observed because we do not find a stable distorted structure for this charge state. This is expected since none of the closely spaced defect states close to the conduction band are occupied. The Re impurity in the +1 state is isovalent to the Mo it replaces, hence both on a qualitative and quantitative point of view, no qualitative change in bonding behavior is expected in line with experimental observations.

In Supplementary Figure 18 the calculated band structure and a selection of frontier $\mathrm{Re}_{\mathrm{Mo}}$ orbitals are shown. For $\mathrm{Re}_{\mathrm{Mo}}^{-1}$ we find a fully occupied orbital $\alpha$ exhibiting a clear two-fold symmetry (Supplementary Figure 18b). An equivalent orbital shape is also found for the $\mathrm{Re}_{\mathrm{Mo}}^{0}$, labelled $\gamma'$. However, this orbital is only singly occupied leading to a spin-polarized band structure and a corresponding unoccupied orbital shape in the orthogonal spin channel ($\beta'$). This orbital is in excellent agreement with experimental $\mathrm{d}I/\mathrm{d}V$ maps shown in Fig. 5b. The positively charged $\mathrm{Re}_{\mathrm{Mo}}^{+1}$ exhibits only three-fold symmetric orbitals as expected, which are in good agreement with the experimental $\mathrm{d}I/\mathrm{d}V$ maps shown in Supplementary Figure 5c.

The prominent three-lobal orbital shape (cf. resonance E in Fig. 5b) could not be observed in the calculations. We suspect that this could in fact be due to a dynamic effect not considered in our calculations: at higher negative bias, inelastic tunnel electrons may drive the transition between the three equivalent distorted geometries that may create a dynamic superposition of several geometric configurations. Following this rationale, we would expect the probed orbital to mimic the superposition of the defect orbital $\gamma'$ with its 120° and 240° copies that may also include the fully symmetric geometry as a transition state in the APES. Qualitative comparison yielded indeed a similar shape to what has been observed experimentally. In-depth molecular dynamics simulations are needed to test this hypothesis and to understand the exact pathways of geometric transitions that may be described as a dynamic JTE.

## SUPPLEMENTARY REFERENCES


[1] Barja, S. *et al.* Identifying Substitutional Oxygen as a Prolific Point Defect in Monolayer Transition Metal Dichalcogenides with Experiment and Theory. *Nat. Commun.* **10**, 3382 (2019).

[2] Schuler, B. *et al.* How Substitutional Point Defects in Two-Dimensional WS2 Induce Charge Localization, Spin-Orbit Splitting, and Strain. *ACS Nano* **13**, 10520–10534 (2019).

[3] Cochrane, K. A. *et al.* Intentional carbon doping reveals CH as an abundant charged impurity in nominally undoped synthetic $WS_2$ and $WSe_2$. *2D Mater.* **7**, 031003 (2020).

[4] Kozhakhmetov, A. *et al.* Controllable p-Type Doping of 2D WSe2 via Vanadium Substitution. *Adv. Funct. Mater.* 2105252 (2021).

[5] Schuler, B. *et al.* Large Spin-Orbit Splitting of Deep In-Gap Defect States of Engineered Sulfur Vacancies in Monolayer WS2. *Phys. Rev. Lett.* **123**, 076801 (2019).

[6] Mitterreiter, E. *et al.* Atomistic Positioning of Defects in Helium Ion Treated Single-Layer MoS2. *Nano Lett.* **20**, 4437–4444 (2020).

[7] Aghajanian, M. *et al.* Resonant and bound states of charged defects in two-dimensional semiconductors. *Phys. Rev. B* **101**, 081201(R) (2020).
